# Supplementary material for: The causal relationship between the human gut microbiota and pyogenic arthritis: a Mendelian randomization study
Source: Front Cell Infect Microbiol. 2024 Nov 26;14:1452480. doi: 10.3389/fcimb.2024.1452480 (PMC11629706; doi:10.3389/fcimb.2024.1452480)
Supplement: Supplementary file 1 [file DataSheet1.zip › mendelian test/ebi-a-GCST90027548.csv_forest.pdf]

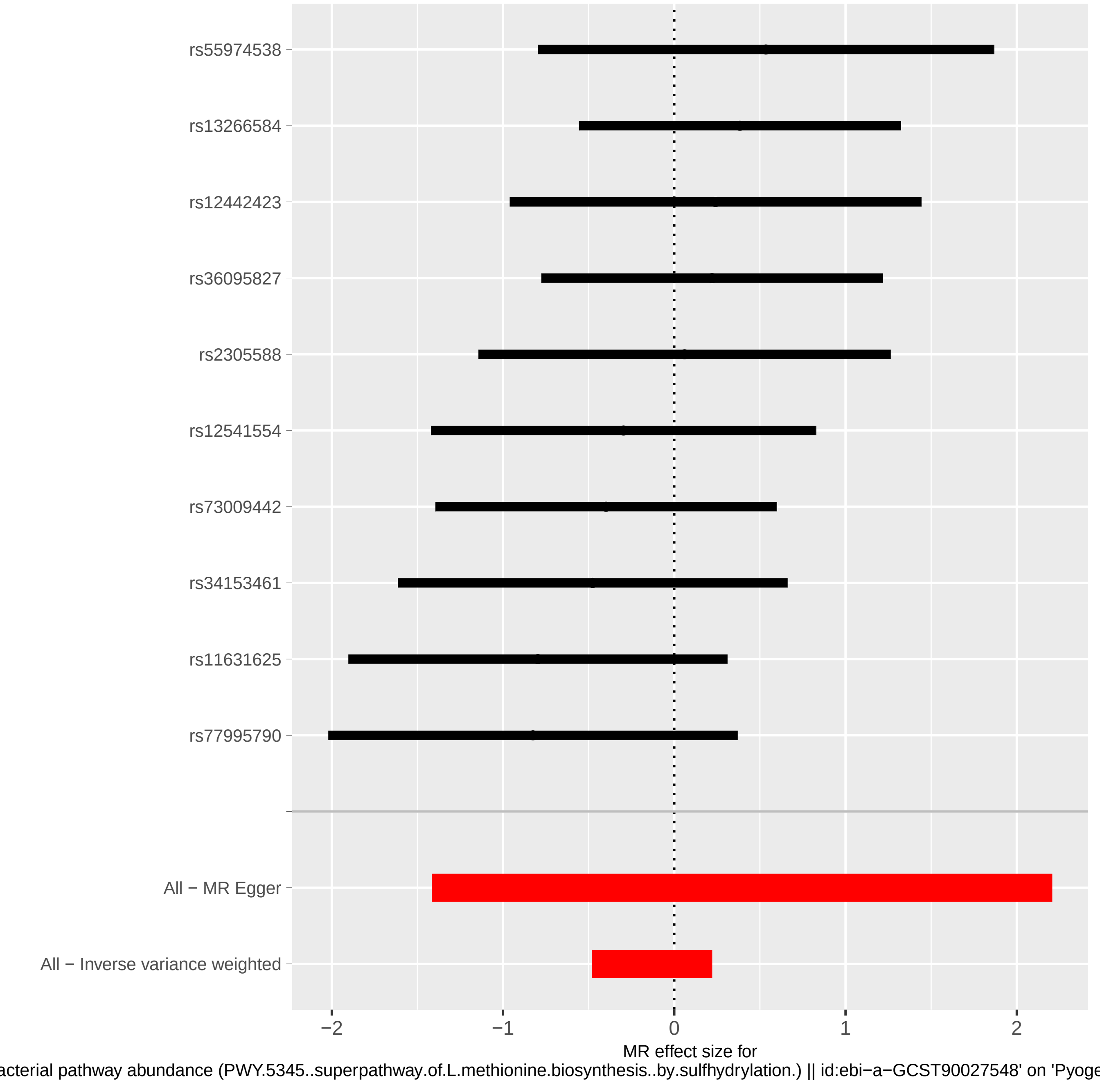

rs55974538
rs13266584
rs12442423
rs36095827
rs2305588
rs12541554
rs73009442
rs34153461
rs11631625
rs77995790
All − MR Egger
All − Inverse variance weighted
−2
−1
0
1
2
MR effect size for
acterial pathway abundance (PWY.5345..superpathway.of.L.methionine.biosynthesis..by.sulfhydrylation.) || id:ebi−a−GCST90027548' on 'Pyoge
